# Changing Emotions About Fukushima Related to the Fukushima Nuclear Power Station Accident—How Rumors Determined People’s Attitudes: Social Media Sentiment Analysis

**DOI:** 10.2196/18662

**Published:** 2020-09-02

**Authors:** Shin Hasegawa, Teppei Suzuki, Ayako Yagahara, Reiko Kanda, Tatsuo Aono, Kazuaki Yajima, Katsuhiko Ogasawara

**Affiliations:** 1 Graduate School of Health Sciences Hokkaido University Sapporo Japan; 2 Quantum Medical Science Directorate National Institutes for Quantum and Radiological Science and Technology Chiba Japan; 3 Hokkaido University of Education Iwamizawa Japan; 4 Faculty of Health Sciences Hokkaido University Sapporo Japan; 5 Department of Radiological Technology Hokkaido University of Science Sapporo Japan

**Keywords:** Fukushima nuclear accident, Twitter messaging, radiation, radioactivity, radioactive hazard release, information dissemination, belief in rumors, disaster medicine, infodemiology, infoveillance, infodemic

## Abstract

**Background:**

Public interest in radiation rose after the Tokyo Electric Power Company (TEPCO) Fukushima Daiichi Nuclear Power Station accident was caused by an earthquake off the Pacific coast of Tohoku on March 11, 2011. Various reports on the accident and radiation were spread by the mass media, and people displayed their emotional reactions, which were thought to be related to information about the Fukushima accident, on Twitter, Facebook, and other social networking sites. Fears about radiation were spread as well, leading to harmful rumors about Fukushima and the refusal to test children for radiation. It is believed that identifying the process by which people emotionally responded to this information, and hence became gripped by an increased aversion to Fukushima, might be useful in risk communication when similar disasters and accidents occur in the future. There are few studies surveying how people feel about radiation in Fukushima and other regions in an unbiased form.

**Objective:**

The purpose of this study is to identify how the feelings of local residents toward radiation changed according to Twitter.

**Methods:**

We used approximately 19 million tweets in Japanese containing the words “radiation” (放射線), “radioactivity” (放射能), and “radioactive substances” (放射性物質) that were posted to Twitter over a 1-year period following the Fukushima nuclear accident. We used regional identifiers contained in tweets (ie, nouns, proper nouns, place names, postal codes, and telephone numbers) to categorize them according to their prefecture, and then analyzed the feelings toward those prefectures from the semantic orientation of the words contained in individual tweets (ie, positive impressions or negative impressions).

**Results:**

Tweets about radiation increased soon after the earthquake and then decreased, and feelings about radiation trended positively. We determined that, on average, tweets associating Fukushima Prefecture with radiation show more positive feelings than those about other prefectures, but have trended negatively over time. We also found that as other tweets have trended positively, only bots and retweets about Fukushima Prefecture have trended negatively.

**Conclusions:**

The number of tweets about radiation has decreased overall, and feelings about radiation have trended positively. However, the fact that tweets about Fukushima Prefecture trended negatively, despite decreasing in percentage, suggests that negative feelings toward Fukushima Prefecture have become more extreme. We found that while the bots and retweets that were not about Fukushima Prefecture gradually trended toward positive feelings, the bots and retweets about Fukushima Prefecture trended toward negative feelings.

## Introduction

### Overview

At 2:46 PM JST (Japan Standard Time) on March 11, 2011, a magnitude-9 earthquake occurred off the Pacific coast of Tohoku—the Great East Japan Earthquake. Its epicenter was off the Sanriku coast, and a tsunami with a run-up height of 14-15 meters followed one hour later, causing a blackout of the Tokyo Electric Power Company (TEPCO) Fukushima Daiichi Nuclear Power Station. A meltdown occurred in reactors 1, 2, and 3 while they were in operation, causing a large quantity of hydrogen to be generated. A hydrogen explosion occurred in reactor 1 on March 12, followed by another explosion in reactor 3 on March 14. Reactors 2 and 4 were damaged, releasing a large quantity of radioactive substances into the environment. This accident was classified as Level 7 according to the International Nuclear and Radiological Event Scale [1], and its effects were evaluated and reported by international organizations [2-4]. They indicated that the accident had increased anxieties about radiation and had given rise to a social phenomenon described as inciting harmful rumors about the disaster area. This has resulted in physical damage from the disaster alongside economic damage from, for example, consumer reluctance to buy agricultural products from the disaster area [5]. In the medical field, anxieties about radiation were reflected in a decrease in the number of computed tomography (CT) scans and other forms of radiographic examination performed on young children in Fukushima Prefecture compared to before the accident [6,7]. One out of every four doctors surveyed reported that parents of young children were refusing to subject them to such examinations due to the risk of radiation [7] and that they had witnessed an aversion to radiation. We believe that people have become gripped by increasingly negative feelings over time concerning Fukushima Prefecture as a disaster area associated with radiation, and this may have influenced behavior such as restrained consumption activities and an aversion to medical radiation.

### Background

Immediately following the earthquake, telephone lines were damaged and communication was cut off or limited. Outgoing calls on mobile phones were restricted up to 95%. For packet communications, NTT (Nippon Telegraph and Telephone) Docomo, the predominant mobile phone operator in Japan, imposed a 30% restriction but it was soon lifted. Other carriers did not implement any restrictions [8]. Therefore, social networking services were used as a means to transmit information, and communities to exchange information rapidly formed on Twitter [9,10]. In a survey, Twitter was found to be the most-used form of social media in coping with the disaster over Facebook or Mixi, and it was shown to have an influence on attitudes toward the Fukushima nuclear accident [11]. Successive reports were issued by the mass media on the condition of the Fukushima nuclear station, the city of Fukushima, other regions affected by radioactive substances, and the effects of the radiation itself; social networks not only carried this reported information and the responses to it, but also rapidly spread unreliable information, misinformation, and ugly rumors, thus indicating that social media can cause social unrest and chaos [12]. Ikegami et al [13] proposed a reliable analysis system for tweets about the 2011 earthquake disaster using topic categories according to latent Dirichlet allocation, with 2960 tweets containing the words “earthquake disaster,” “earthquake,” “tsunami,” “radioactivity,” “radioactive substances,” and “Becquerel” as a dataset, and sentiment analysis using a semantic direction dictionary [14]. Wang and Kim [15] showed that behavior in cyberspace and real-world behavior mutually influence one another. Using this as a basis, we believe that people who have come into contact with social anxieties and ugly rumors as spread on social media networks have an increased aversion to Fukushima Prefecture, which may lead to additional harmful rumors and a refusal of radiographic examinations.

### Prior Work

It has been shown that 5 years after the accident, groups that relied on the internet as a source of information had significantly higher anxieties about health issues caused by radiation exposure than groups that used other information sources [16]. Mothers of children younger than elementary school age using Twitter and other forms of social media were shown to have a higher degree of risk perception and to actively pursue risk-reducing activities [17]. It is not hard to imagine that high anxiety and risk-reducing activities may lead to a refusal of radiographic examinations of children. Risk communication is valuable in eliminating these social anxieties, but studies have indicated that social media was not fully utilized at the time of the Fukushima accident [18]. Yagahara et al [19] analyzed tweets for 7 days from the day of the earthquake on March 11 until March 17 to examine the changes in interest in radiation among Japanese citizens. Their analysis was based on co-occurrence networks related to radiation as the accident’s situation progressed. Since the analysis was also conducted in relation to regions, it did not identify how the interest subsequently formed people’s attitudes toward Fukushima. Aoki et al [20] surveyed tweeting trends 1 year after the earthquake by dividing regions tweeted about into four zones based on geotags. This analysis only concerned the regions that people were tweeting from and did not analyze the content of the tweets themselves. Therefore, we believe that surveying how people received information associated with the Fukushima nuclear accident and how they reacted to it will be of great significance in establishing a basis for effective risk communication when similar disasters and accidents occur in the future.

### Goal of This Study

This study concerns the decrease in CT scans and other radiographic examinations [6,7], the persistent restrained buying of agricultural products by consumers, and harmful rumors [5] regarding Fukushima Prefecture. Its purpose is to comparatively identify how feelings associated with radiation have shifted from Fukushima Prefecture to other regions 1 year after the 2011 earthquake and accident and thereby clarify how such situations are formed. People may have become gripped by more negative feelings over time regarding Fukushima Prefecture as a disaster area associated with radiation, which may have influenced an increased aversion toward Fukushima Prefecture in general.

## Methods

### Overview

In this study, we selected statements about radiation containing the words “radiation” (放射線), “radioactivity”（放射能), and/or “radioactive substances” （放射性物質） that were posted on Twitter in Japanese between the occurrence of the Great East Japan Earthquake at 12 AM JST on March 11, 2011, until 1 year later (ie, 11:59 PM JST on March 10, 2012) using approximately 19 million tweets. We grouped tweets starting with retweets (RTs) and quote tweets (QTs) to indicate that they were automatic tweets—referred to as *bots*—and RTs that had reposted someone else’s tweet into an RT group, leaving approximately 9 million tweets, excluding the RT group, as a target group. Bot accounts were accounts in which the user’s ID began or ended with the word *bot*. In order to more accurately sample the original poster’s feelings, we deleted anything in the target group that followed an RT, which indicated that someone else’s tweet had been reposted, and then processed each of the tweets using semantic orientation, which refers to a binary attribute that shows how a word might generally carry a positive or a negative impression. Takamura et al [14] used the Japanese dictionary *Iwanami Kokugo Jiten* to create semantic orientation values of words by assigning semantic orientation values with actual values from –1 (carries mostly a negative impression) to 1 (carries mostly a positive impression) for 49,002 nouns, 4254 verbs, 665 adjectives, and 1207 adverbs. All words and phrases contained in this study’s tweets were scored using the above morphological analysis based on the original form of the words by using their semantic orientation values. In addition, the data used tweets containing any of the following words: “radiation,” “radioactivity,” or “radioactive substances.” The semantic orientation values for these words were evaluated: “radioactivity” had a value of –0.598318, “radiation” had a value of –0.560393, and “radioactive” had a value of –0.178744; there were no instances of “radioactive substances” in Takamura et al’s correspondence table. These were originally logged as negative words. The purpose of this study is to analyze the feelings associated with the three words above; in order to exclude these influences, we scored these words as having 0 points. We scored the words that were not included in Takamura et al’s semantic orientation values of words [14] in the correspondence table as having 0 points as well, so as to avoid affecting the tweets’ semantic value orientations, which we calculated using the following formula:

*T_pn_* = ∑*W_pn_*/*W_c_*

where T_pn_ represents the tweet semantic orientation value, W_pn_ represents the word semantic orientation value, and W_c_ represents the number of words contained in a tweet.

In order to categorize the prefectures to which the tweets related, the words and phrases related to regions contained in tweets (ie, place names, telephone numbers, and postal codes) were sampled and categorized by prefecture. For place names, we morphologically analyzed tweets using the morphological analysis engine MeCab [21] and the mecab-ipadic-NEologd [22] dictionary and searched the following word types for their address character strings with the Yahoo! Geocoding application programming interface [23]: parts of speech = nouns; parts of speech (subcategory 1) = proper nouns; and parts of speech (subcategory 2) = regions. We then identified and categorized the prefectures (Multimedia Appendix 1 contains the scripts to process these procedures).

### Sentiment Analysis About Radiation Regarding Fukushima Prefecture and Other Prefectures

**Figure 1:**
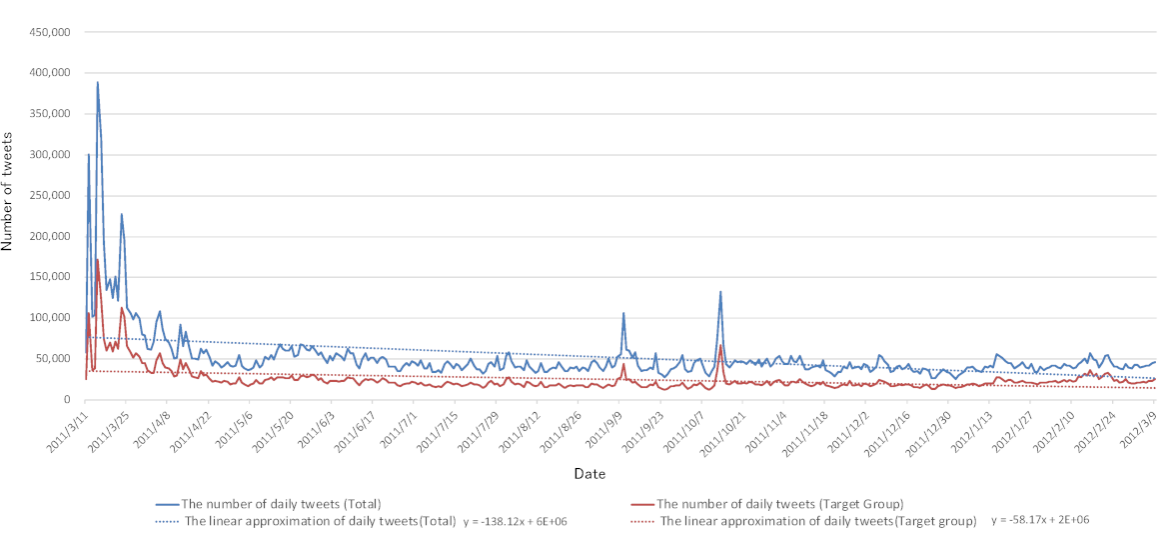
The number of tweets per day.

**Figure 2:**
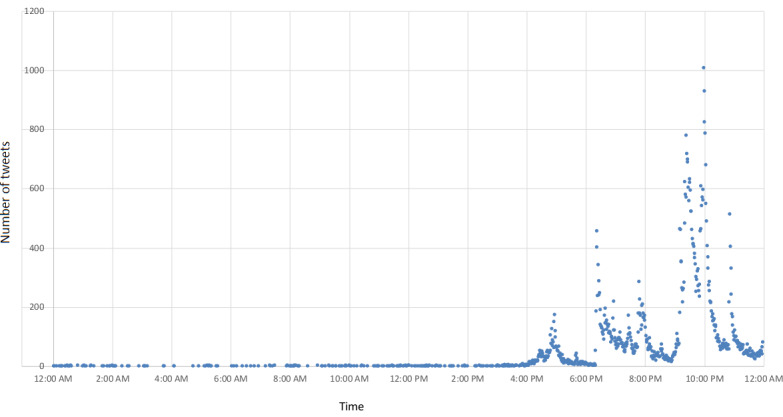
The number of tweets by the minute on March 11, 2011.

The tweets categorized by prefecture were divided into two groups: Fukushima Prefecture and other prefectures. We sampled the average weekly tweets’ semantic orientation values for Fukushima Prefecture and other prefectures and then surveyed how the feelings on radiation regarding each region had changed. The dataset used 18,841,755 out of 18,851,259 tweets of words having semantic orientation values. Semantic orientation values were sampled for almost all tweets. The dataset included a total of 18,851,259 tweets and a target group of 9,025,831 tweets, excluding bots and RTs. The respective daily changes in the number of tweets are indicated in Figure 1 by a blue line and a red line. The linear approximation of each is drawn with a dashed line. Figure 2 shows the daily number of tweets at a more granular level (ie, by the minute) for March 11, the day the Great East Japan Earthquake occurred.

**Figure 3:**
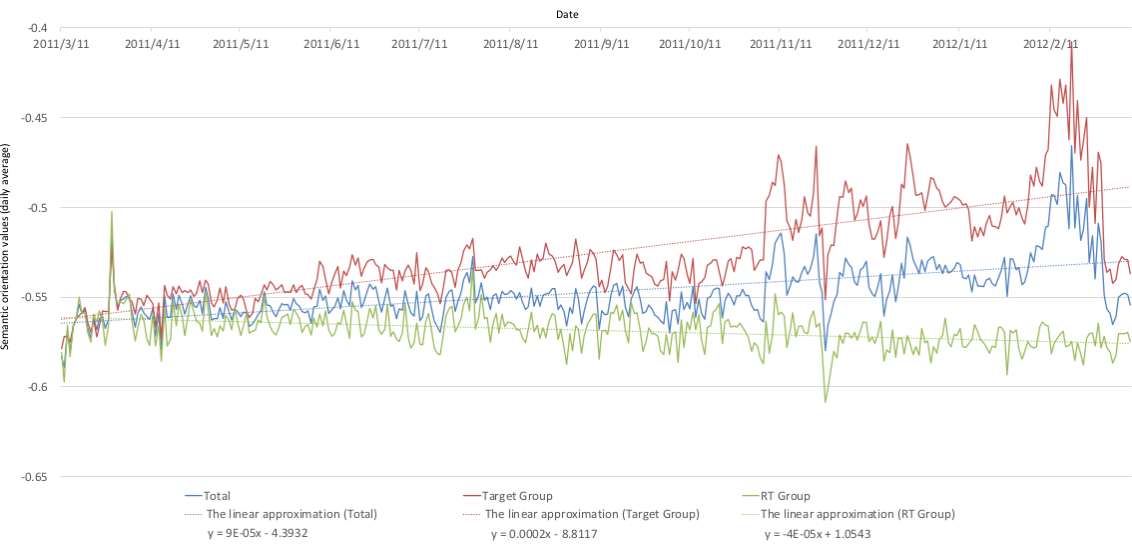
Daily average of tweets' semantic orientation values. RT: retweet.

**Figure 4:**
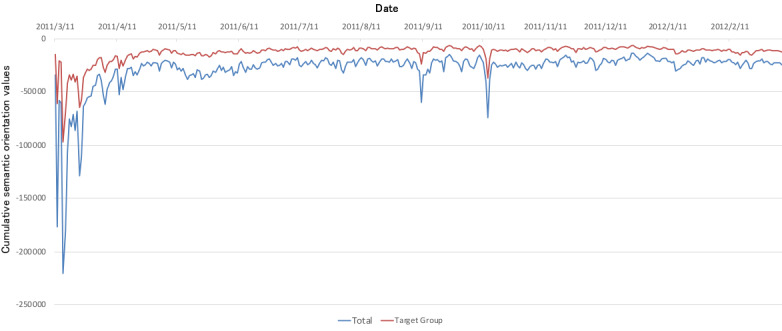
Daily integration of semantic orientation values.

**Figure 5:**
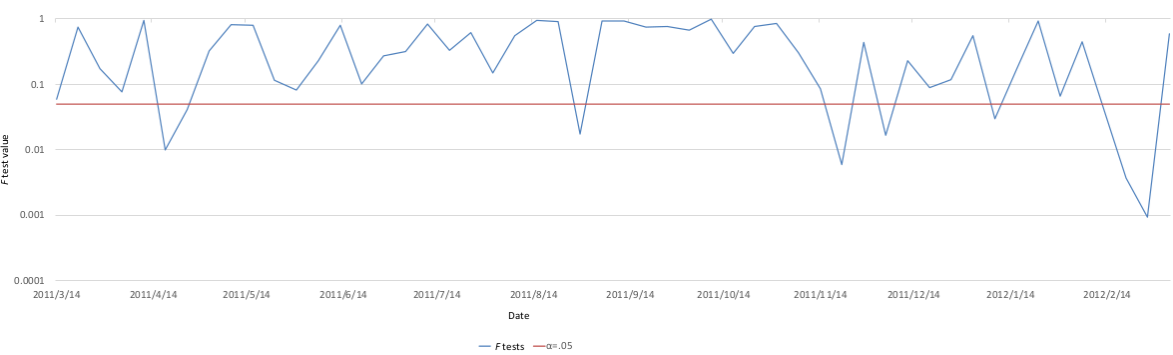
*F* test for target and retweet (RT) groups by week.

Figures 3 and 4, similar to Figure 1, show the respective daily average changes and cumulative changes in the tweets’ semantic orientation values. In addition, average changes in the RT group’s semantic orientation values are included in Figure 3, and the linear approximation is represented by a dashed line. Figure 5 shows the changes during weekly *F* tests of the average semantic orientation values in order to observe a correlation between the target group and the RT group. A red line is drawn using the significance level α=.05.

**Figure 6:**
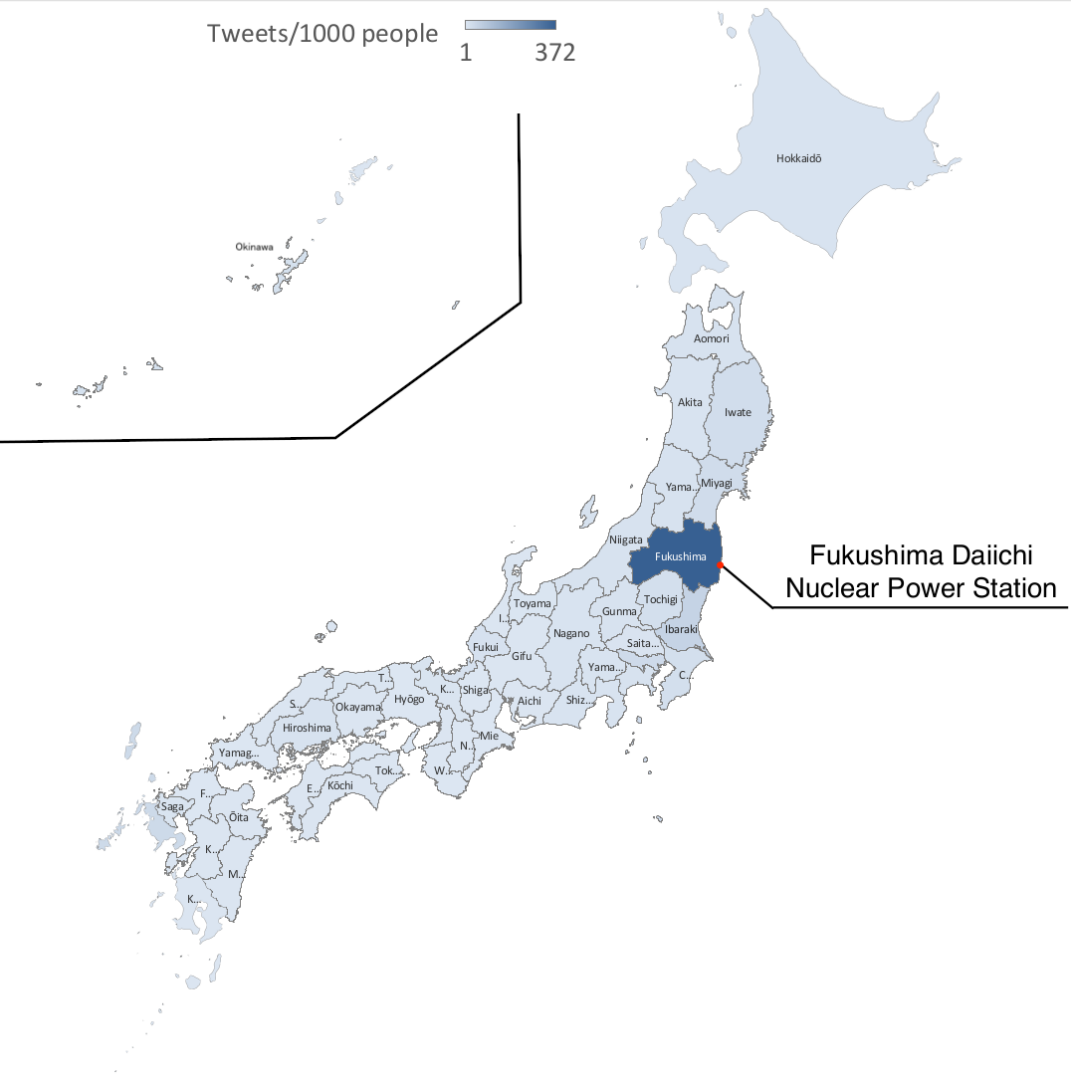
The number of tweets per 1000 people.

The target group was categorized by prefecture. There were 34,233 words representing regional identifiers and 3,004,726 tweets in the target group containing words representing the regional identifiers. We calculated the number of tweets per 1000 people according to the breakdown in each prefecture and its population as of October 1, 2011 [24]. This is represented by shading on the map in Figure 6. Details are shown in Table 1, in which the *Other* row contains words that made it difficult to identify the prefecture and foreign place names. Words that made it difficult to identify the prefecture include universal words representing addresses, such as “1-chome” and “1-banchi,” for example. Foreign place names frequently included regions that experienced nuclear power accidents in the past, such as Chernobyl and Three Mile Island.

**Table 1:** Breakdown of words and tweets by prefecture.

Prefecture	Number of words representing the area in the prefecture	Number of tweets	Tweets per 1000 people
Hokkaido	635	51,871	9
Aomori	158	18,181	13
Iwate	545	32,319	25
Miyagi	911	64,714	28
Akita	200	10,473	10
Yamagata	295	13,523	12
Fukushima	1535	741,178	372
Ibaraki	1013	155,482	53
Tochigi	650	41,832	21
Gunnma	714	26,892	13
Saitama	1647	55,702	8
Chiba	1757	129,784	21
Tokyo	2614	441,874	33
Kanagawa	1766	108,510	12
Niigata	380	29,238	12
Toyama	100	4919	5
Ishikawa	121	3461	3
Fukui	137	11,437	14
Yamanashi	313	7881	9
Nagano	663	21,466	10
Gifu	325	8582	4
Shizuoka	615	36,133	10
Aichi	696	21,380	3
Mie	208	2339	1
Shiga	131	3497	2
Kyoto	319	18,232	7
Osaka	677	35,824	4
Hyogo	362	9341	2
Nara	150	3208	2
Wakayama	115	2271	2
Tottori	71	2774	5
Shimane	79	3081	4
Okayama	134	5937	3
Hiroshima	195	35,599	12
Yamaguchi	109	2462	2
Tokushima	75	2113	3
Kagawa	77	2229	2
Ehime	105	3974	3
Kochi	99	2596	3
Fukuoka	326	12,243	2
Saga	83	5757	7
Nagasaki	125	48,477	34
Kumamoto	123	4620	3
Oita	104	3590	3
Miyazaki	106	3284	3
Kagoshima	127	4559	3
Okinawa	165	22,115	16
Other	16,235	1,396,553	N/A^a^

^a^N/A: not applicable; this was not calculated, as the population size for this category is not known.

**Figure 7:**
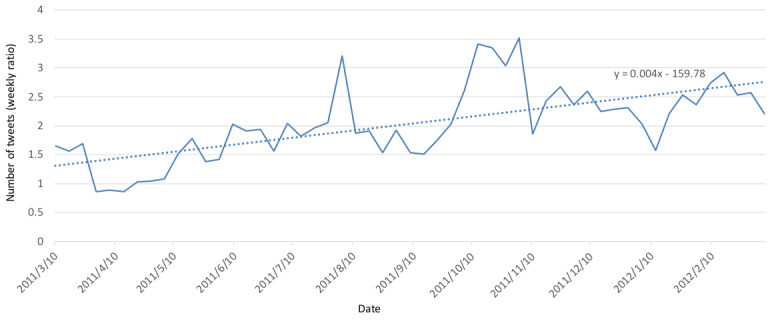
Weekly ratio of the number of tweets for Fukushima and other prefectures. The dotted line represents the linear approximation.

**Figure 8:**
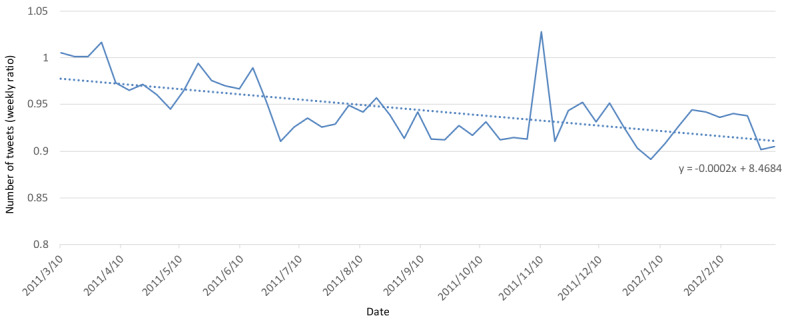
Weekly average ratio of semantic orientation values for Fukushima and other prefectures. The dotted line represents the linear approximation.

**Figure 9:**
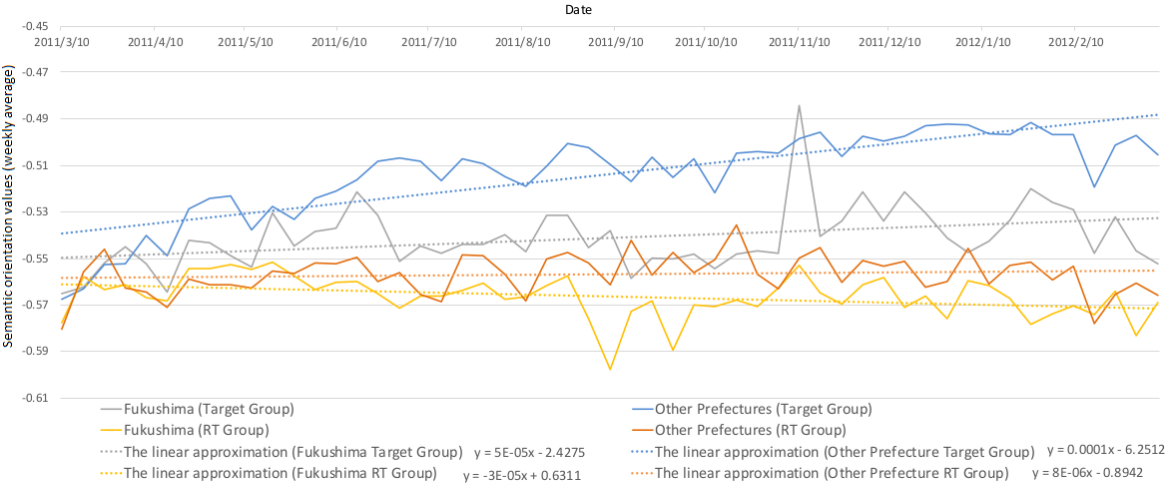
Weekly average of semantic orientation values for Fukushima Prefecture and other prefectures. RT: retweet.

Figures 7 and 8 show the ratio of the number of tweets between the target group in Fukushima Prefecture and other prefectures as well as the ratio of the average semantic orientation values. Semantic orientation values ranged from –1 to 1; thus, the ratio of average values in Figure 7 is the ratio when 1 is added to the average of the semantic orientation values and, therefore, it results in a value between 0 and 2. Figure 9 shows the changes in the average values of the semantic orientations for Fukushima Prefecture and other prefectures for the target group and RT group; details of the plots are as follows:

1. The solid grey line represents the weekly average of semantic orientation values, excluding bots and RTs, in Fukushima Prefecture.
2. The solid blue line represents the weekly average of semantic orientation values, excluding bots and RTs, outside of Fukushima Prefecture.
3. The solid yellow line represents the weekly average of semantic orientation values of bots and RTs in Fukushima Prefecture.
4. The solid orange line represents the weekly average of semantic orientation values of bots and RTs outside of Fukushima Prefecture.

The linear approximation was drawn with a dashed line for each respective line.

## Results

### Overview

First, we discuss the characteristics and trends in the overall dataset. After discussing the characteristics for each prefecture, we then compare them with Fukushima Prefecture—as the disaster area and the main subject of harmful rumors—and other prefectures.

### Characteristics and Trends in the Overall Dataset

Table 2 shows the table of events in 2011 in chronological order. At 2:46 PM JST on March 11, 2011, the Great East Japan Earthquake occurred, and a resulting tsunami struck various locations about one hour later. This gave rise to concerns about damage to the nuclear power stations on the Pacific coast at around 4 PM. Previously, there had been a few tweets containing the words “radiation,” “radioactivity,” and “radioactive substances,” but we found that the tsunami resulted in a rapid increase in tweets containing these three words (see Figure 2). Similar to tweets that were trending at the time of the 2010 Chilean earthquake as analyzed by Mendoza et al [25], many of the tweets posted immediately after the Great East Japan Earthquake can be found in our dataset. The overall amount of tweeting consisted of approximately 8% (of all tweets in the 1-year period) being posted 1 week after the earthquake and approximately 21% being posted 1 month after the earthquake, and there is a subsequent and gradually decreasing trend for the remaining period. As shown in Figure 1, approximately 300,000 tweets were posted on March 12 when reactor 1 experienced a hydrogen explosion, and approximately 400,000 tweets were posted on March 15 when the damage to reactors 2 and 4 became clear. On March 23, thyroid equivalent dose predictions involving iodine-131 for infants (under 1 year old) using the System for Prediction of Environmental Emergency Dose Information were released by the Cabinet Office’s Nuclear Safety Commission [26], and approximately 230,000 tweets were posted. Thereafter, tweeting hovered around 50,000 per day.

On September 10, the then-Minister of Economy, Trade, and Industry reportedly resigned after a visit to the exclusion zone of the Fukushima nuclear disaster, where he joked to a journalist, “I’ll give you radiation.” Later, a fire broke out at Sendai Nuclear Power Plant, and the information about these two events spread. On October 14, the number of tweets exceeded 100,000 in response to news that a radium ray source was found under the floor of a private home in Setagaya Ward. As shown in Figure 3, many negative tweets were posted immediately after the Great East Japan Earthquake, and as shown by the approximation curve, they gradually trended positively thereafter. However, we found they were under –0.4 overall, and tweets expressing negative feelings about “radiation,” “radioactivity,” and “radioactive substances” were still posted. Although there is no difference between the overall dataset (see Figure 3, blue line) and most of the semantic orientation values in the target group immediately after the earthquake, the target group tended to be faster in its acceleration from negative to positive. More negative tweets were posted by bots and RTs, and we found that the sentiments in the bots and RTs tended to be negative as shown by the negative slope of the approximation’s straight line. Bots and RTs often serve as a means for information to be spread widely. However, they were found to spread information containing negative sentiments more easily. This suggests that bots and RTs sensitize and lead users and communities that encounter this information to an increased aversion toward certain things.

There were characteristic changes in semantic orientation values close to the dates given below. These have been included, together with how much content was spread on these days. On March 28, a website visualizing the environmental radioactivity levels all over Kanto was launched [27]. Knowledge of the website was rapidly spread as a means of information sharing, together with positive words describing the website as easy to understand, so it tended to score positively at 0.029-0.067 points both before and after its launch, and the same trend was shown in both groups.

On November 11, a large number of more positive tweets than average were posted stating “Sign an emergency petition to save the children of Fukushima” (semantic orientation value: –0.396); thus, the tweets largely trended positively. These tweets were not posted by bots and were not in the form of RTs, and we found that the divergence between the groups was significant in the weeks before and after, as shown in Figure 4.

On November 23, Geiger counter advertisements, with semantic orientation values of about 0.05-0.40, were posted approximately 2-3 times as often compared to the days before and after—November 22 saw 1797 out of 34,173 tweets (5.26%); November 23 saw 3468 out of 33,778 tweets (10.27%); and November 24 saw 1087 out of 40,348 tweets (2.69%)—and this was believed to be influential. Similar trends were confirmed for the December 24 peak of the Geiger counter advertisements both before and after.

On November 26, it was reported that TEPCO had responded on November 24, “Any radioactive substances were not the property of TEPCO. Consequently, TEPCO has no responsibility for decontamination” [28]. However, this information was spread using negative words and, as a result, the semantic orientations in both groups largely trended negatively.

Interestingly, regardless of whether similar lines were previously drawn for the target group and the RT group, from December 7 to February 27, divergence emerged in the form of changes between the two groups. Particularly in the target group, the periods from December 21 to January 15 and from February 7 to February 27 peaked positively. On December 18, a maximum of –0.41 was reached, but no corresponding peak was formed in the RT group, which hovered around –0.58. As with the weekly *F* tests of the target group and RT group as shown in Figure 4, a significant divergence was seen between the groups during this period.

**Table 2:** Chronological listing of events in 2011.

Date (year/month/day)	Event and details
2011/03/11	An earthquake off the Pacific coast of Tohoku occurred.
2011/03/12	Reactor 1 of Fukushima Daiichi Nuclear Power Station experienced a hydrogen explosion.
2011/03/15	The damage to reactors 2 and 4 of Fukushima Daiichi Nuclear Power Station became clear.
2011/03/23	Thyroid equivalent dose predictions involving iodine-131 for infants (under 1 year old) using the System for Prediction of Environmental Emergency Dose Information were released by the Cabinet Office’s Nuclear Safety Commission.
2011/03/28	A website visualizing the environmental radioactivity levels all over Kanto was launched.
2011/09/10	The then-Minister of Economy, Trade, and Industry reportedly resigned after a visit to the exclusion zone of the Fukushima nuclear disaster, where he joked to a journalist, “I’ll give you radiation.” A fire broke out at Sendai Nuclear Power Plant.
2011/10/14	A radium ray source was found under the floor of a private home in Setagaya Ward, Tokyo.
2011/11/11	A large number of tweets were posted stating “Sign an emergency petition to save the children of Fukushima.”
2011/11/23	Geiger counter advertisements were posted approximately 2-3 times as often compared to the days before and after.
2011/11/24	At Tokyo District Court, TEPCO responded, “Any radioactive substances were not the property of TEPCO. Consequently, TEPCO has no responsibility for decontamination.”
2011/11/26	TEPCO's response was reported in the press.

### Characteristics Per Prefecture

As shown in Table 1, there was a spike in tweets about Fukushima Prefecture as a disaster area, which stands out in terms of population ratio. Areas in east Japan close to the disaster area had a larger number of tweets than west Japan and were at high rates in terms of their population ratio. Osaka and Kyoto, which are major cities in west Japan, had high numbers of tweets but were at the same level as other regions in west Japan in terms of the population ratio. This suggests that this bias does not affect the data on Twitter, which has more users in urban areas. In addition, Hiroshima Prefecture and Nagasaki Prefecture, which were devastated in the past by the atomic bomb, stood out in tweeting from other regions in west Japan, suggesting that people may be recalling place names associated with radiation. In the case of Okinawa Prefecture, the US military base located there has been alleged to possess nuclear weapons many times in the past and seemed to be the name of a place brought up in connection to radiation.

### Comparison of Fukushima Prefecture and Other Prefectures

As shown in Figure 7, the ratio of the number of tweets about Fukushima Prefecture and other prefectures shows an increasing trend, and the interest in Fukushima Prefecture has risen. Including other prefectures, the number of tweets was at 3 times the highest amount, and the right intercept of the linear approximation was at 2.5 times the highest amount.

The ratio of the average of the semantic orientation values for Fukushima Prefecture and other prefectures fell roughly below the value of 1 when unprecedented periods were excluded. As drawn by collinear approximation, the ratio gradually fell, which may be interpreted as the deepening of negative feelings about Fukushima Prefecture when compared to other regions. The weekly tweet number ratio on November 10 had not increased much compared to the previous week, but the semantic orientations about Fukushima largely trended positively. This is believed to have been influenced by the larger number of more positive tweets than average being spread stating “Sign an emergency petition to save the children of Fukushima” (semantic orientation value: –0.396). These tweets were not posted by bots or in the form of RTs.

As shown in Figures 1 and 3, the overall number of tweets decreased and, as a result, the overall semantic orientation values about radiation trended positively. However, as shown in Figures 7 and 8, the ratio of the number of tweets about Fukushima Prefecture compared to other prefectures increased, and it is thought that the decreasing ratio of semantic orientation values indicates that emotions sharply trended negatively toward Fukushima Prefecture in regard to radiation.

In Figure 9, we found that the RT group had more posts about negative feelings compared to the target group, as in the discussion of Figure 3 above. However, it is possible to confirm the same trend in tweets about regions. Surprisingly, while other tweets trended toward positive feelings, only the collinear approximation of the average trend in the semantic orientation values for the RT group relating to Fukushima Prefecture showed a negative trend, with the tendency to be broadcast with increasing negative feelings over time. This strongly suggests that bots and RTs disperse information that has more negative emotions, and users who come into contact with this information perceive matters relating to the radiation in Fukushima Prefecture with negative emotions, leading to an increased aversion among these people toward Fukushima Prefecture. These results support the hypothesis that people have become gripped by more negative feelings over time regarding Fukushima Prefecture as a disaster area associated with radiation, which may have influenced an increased aversion toward Fukushima in general.

## Discussion

### Principal Findings

This study was a unidirectional survey of the feelings that nationwide Twitter users had about each prefecture and radiation. It is felt that residents’ feelings toward a particular region, which is subject to harmful rumors, are important when elucidating and ameliorating the processes by which people’s aversions to Fukushima are increasing. The purpose of this study was to identify how information on radiation changed 1 year after the Fukushima nuclear accident with regard to different regions in Japan. We found that immediately after the accident, negative feelings about radiation trended positively over time, but bots and RTs were slow to do so compared to other tweets. We found that tweets associating Fukushima Prefecture with radiation clearly showed more negative feelings than those about other prefectures on average; they further trended as negative and increased in percentage over time. Tweets about radiation decreased overall, and feelings about radiation also trended positively. However, the fact that tweets about Fukushima Prefecture trended negatively while rising in percentage suggests that negative feelings toward Fukushima Prefecture were intensifying. We found that while the bots and RTs that were not about Fukushima Prefecture gradually trended toward positive feelings, the bots and RTs about Fukushima Prefecture trended toward negative feelings. These results point to the possibility that people’s aversions toward Fukushima Prefecture increased as a result of negative feelings that associate Fukushima Prefecture with radiation, as spread by bots and RTs. Signals about risk, such as health risks from radiation, are often amplified by individual and social processes, such as cultural groups and interpersonal networks, that amplify people's responses [29]. This supports the hypothesis that people have become gripped over time by a more negative impression of Fukushima Prefecture as a disaster area associated with radiation, which may have influenced an increased aversion toward Fukushima in general. To confirm this, tracking interaction between a bot and a person at an individual level should be performed as a next step. Additionally, it is well known that confirmation bias is amplified by the use of filter bubbles on social media [30,31]. This effect should be taken into account to analyze the impact of RTs and bots on people’s aversions.

### Limitations

Gore et al showed that there can be significant geographic bias in the sentiment expressed in tweets over the same time period [32]. Padilla et al showed that the sentiment expressed in tweets can be biased based on whether people are local or visiting an area and what other activities they have completed during the course of a day [33]. This study did not take this into account, as it conducted a one-way sentiment analysis of emotions directed toward Fukushima. In the future, in order to identify the process by which people’s aversions increase, we want to clarify the feelings that people in a particular region have toward regions that are subject to harmful rumors, such as Fukushima. In a survey by Aoki et al [20], geotagged tweets made up only 0.25% of the target data; therefore, more comprehensive data are required. In addition, since there are deviations in the age composition and region of Twitter users’ residences, the users are not necessarily representative of the nation as a whole. In this study, we determined the semantic orientation of tweets according to semantic orientation values toward words. Thus, the correct semantic orientation of tweets is not necessarily representative in terms of the context within tweets or the context based on their relationship to preceding and following tweets. Clearly sarcastic statements like “radioactivity is delicious” have positive semantic orientations due to the word “delicious,” so these tweets are judged to have positive semantic orientations. It seems a technique is needed for correctly evaluating the semantic orientation in terms of both the written sentences and their context. It is well known that there is a normalcy bias in the responses of the public figures to a serious event that has occurred suddenly and unexpectedly [34]. In particular, when a severe nuclear accident occurs, affected people may very likely tweet simply to calm their own minds. As a result, their tweets may reflect not their feelings but their wishes. Additionally, in some cases, recall bias has led to an overestimation of the health risks from radiation, and tweets then expressed excessive aversion. In order to analyze the impact of these cognitive biases [35], it is necessary to evaluate the content of the tweet and the network.

Japanese speakers tend to skip words if their meaning is conveyed [36], which is significant since Twitter is limited to 140 characters. “Radioactive iodine” and “radioactive cesium” are frequently used radioactive isotopes that are often indicated simply with “iodine” and “cesium.” “Cesium” is not a familiar word in daily life and likely indicates the radioactive isotope cesium. Further, feelings related to words that are often omitted when discussing radiation need to be surveyed instead of just “radiation,” “radioactivity,” and “radioactive substances.”

Whether a tweet is from a bot is determined based on whether the term *bot* is found before or after the user’s ID. Therefore, all bots cannot be accurately identified. In addition, we believe that tweets from accounts that repeatedly post the same information that are not advertising accounts, that do not follow this format, or that are not RTs need to be surveyed as part of the RT group or split off into a separate group. However, considerable effort is required to look up and verify each tweet one by one. Communities are formed by Twitter’s *follow* function, and sharing and propagating information occurs through RTs.

In the future, it may be important to elucidate the process by which people’s attitudes become fixed through a survey of how information is propagated by community networks and RTs as well as the feelings that people become gripped by when an aversion to Fukushima increases.

## Appendix

Multimedia Appendix 1 The scripts used in this research. analyze_store_tweets_area_name.py: get area names in tweets. geocoder.py: get full address from area names by yahoo geocoder API. Thus, it can get prefecture name. calc_tweets_SOV.py: calculate the semantic orientation value of each tweets.

## Abbreviations

CT: computed tomography
JST: Japan Standard Time
NTT: Nippon Telegraph and Telephone
QT: quote tweet
RT: retweet
TEPCO: Tokyo Electric Power Company
